# Computerized tomography based tumor-thickness measurement is useful to predict postoperative pathological tumor thickness in oral tongue squamous cell carcinoma 

**DOI:** 10.1186/s40463-015-0089-z

**Published:** 2015-11-16

**Authors:** J. Madana, Frederick Laliberté, Grégoire B. Morand, Deeke Yolmo, Martin J. Black, Alex M. Mlynarek, Michael P. Hier

**Affiliations:** Department of Otolaryngology-Head and Neck Surgery, Sir Mortimer B. Davis-Jewish General Hospital, McGill University, 3755 Côte Ste-Catherine Road, Montreal, QC Canada H3T 1E2

**Keywords:** Tumor thickness, Oral tongue, Computerized tomography, Measurement, Postoperative

## Abstract

**Background:**

Tumor thickness has been shown in oral tongue squamous cell carcinoma (OTSCC) to be a predictor of cervical metastasis. The postoperative histological measurement is certainly the most accurate, but it would be of clinical interest to gain this information prior to treatment planning. This retrospective study aimed to compare the tumor thickness measurement between preoperative, CT scan, and surgical specimens .

**Methods:**

We retrospectively included 116 OTSCC patients between 2001 and 2013. Thickness was measured on computer tomography imaging and again surgical specimens.

**Results:**

The median age was 66 years. 62.8 % of patients were smokers with a mean of 31.4 pack-years. Positive nodal disease was reported in 41.2 %. Mean follow-up time was 33.1 months. The correlation between CT scan-based tumor thickness and surgical specimens based thickness was significant (Spearman rho = 0.755, *P* < 0.001).

**Conclusion:**

Tumor thickness assessed by CT scan may provide an accurate estimation of true thickness and can be used in treatment planning.

## Introduction

Oral squamous cell cancer (OSCC) accounts for approximately 2.5 % of all cancers in the United States and are primarily associated with chronic consumption of alcohol and tobacco [1]. OTSCC are particular in their high propensity to metastasize given the rich lymphatic drainage among most of the anatomic subsites. This is clinically relevant as cervical lymph node metastasis are thought to be the single most important prognostic factor in head and neck carcinoma, with certain studies reporting an associated 5-fold increase in mortality [2].

One issue is that a high proportion of OSCC present with occult cervical metastasis. The literature indeed shows that they are present in 18 % to 53 % of cT1-2 N0 oral cavity cancers [2–5]. The prediction and therapeutic assessment of occult metastasis is important, as nodal metastasis that become clinically apparent have been shown to be harder to treat. [6, 7]. Given the lack of sensitivity of current imaging modalities to detect micrometastases, many patients with early OCSCC will undergo elective neck dissections. This approach will be useful for the proportion of patients with occult metastasis but amounts to overtreatment in patients without.

Analogous to melanoma [8], tumor thickness is now increasingly used in OSCC.to predict lymph node metastasis [9–13]. While the gold standard to measure tumor thickness is ultimately postoperative tumor thickness assessed on definitive pathology, it would be useful to establish a method to evaluate tumor thickness pre-operatively, thus avoiding two-step surgery. Different modalities, allowing preoperative measurement of tumor thickness are being evaluated with conflicting results.

Our retrospective study evaluates the accuracy of computer tomography (CT scan) in predicting histological tumor thickness.

## Materials and methods

### Patient population

Records of patients who presented to the Jewish General Hospital with a new diagnosis of oral tongue SCC during the period of January 2001-January 2013 were retrieved (*n* = 116). Information was collected retrospectively on patients’ characteristics (i.e. age, gender, smoking and drinking habits), clinical and pathological tumor characteristics (i.e. TNM staging, tumor thickness according to CT scan, histological postoperative tumor depth/thickness). CT scan reports were reviewed for all patients to collect the tumor thickness data. Likewise the final postoperative reports (signed out by Head and Neck Pathologists) were reviewed to obtain data regarding surgical tumor thickness.

### Statistical analysis

Binary variables were associated in contingency tables using the two-sided Fisher exact test. Odds ratio (OR) and 95 % confidence interval (95%CI) were calculated also using two-by-two tables, according to the Mantel-Haenszel method. A *P* value lower than 0.05 was considered to indicate statistical significance. Non-parametric Spearman method was used to assess correlation between numeric scaled variables. Statistical analyses were performed using SPSS® 21.0.0 software (IBM©, Armonk, NY, USA).

## Results

### Study population

In total, 116 patients were included in the study. The median age was 66 years (range 28–92). There were 50 females (43.1 %) and 66 (66.9 %) males. Alcohol consumption was reported in 42.2 % of the cases. Regarding smoking habits, 37.2 % of patients were never smokers, 35.1 % former smokers and 27.7 % current smokers. The mean reported cumulative cigarette consumption was 31.4 ((±S.E. 2.4) pack years. Pathological positive nodal disease was reported in 41.2 %. Clinical stage I, II, III and IV were reported in 29.8 %, 16.7 %, 15.8 % and 32.5 %, respectively. Stage 0, corresponding to *in situ* tumor was reported in 5.3 %. Mean follow-up time was 33.1 months (±S.E. 3.7). Clinically negative necks turned out pathologically positive in 23.4 % patients. Locoregional recurrence was reported in 22.6 % of the patients, while 13.6 % of them suffered disease-specific death. Overall, 18.1 % died of any cause in this cohort.

### CT tumor thickness versus postoperative tumor thickness

**Fig. 1:**
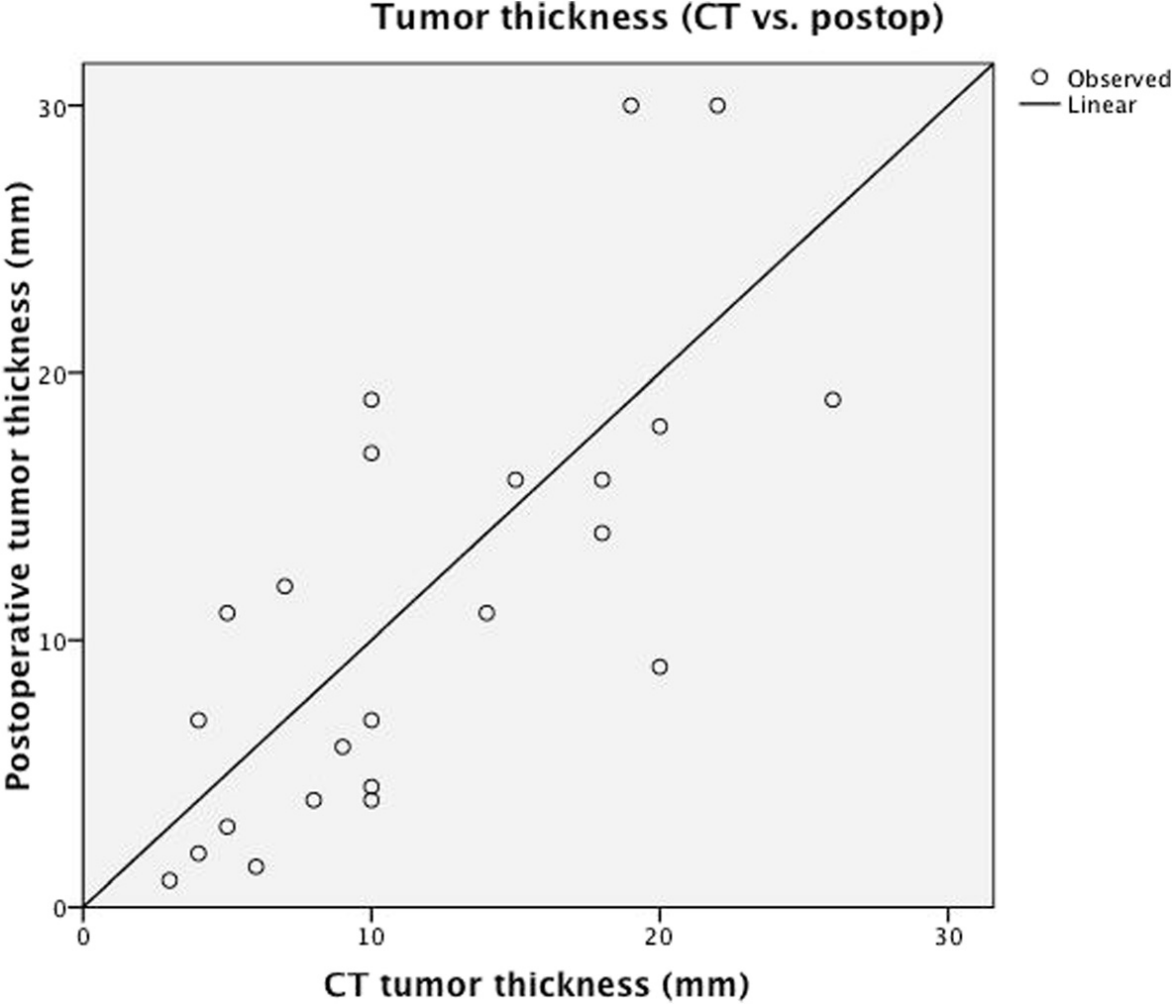
Shows a significant correlation between CT thickness and postoperative thickness (Spearman rho = 0.755, *P* < 0.001)

**Fig. 2:**
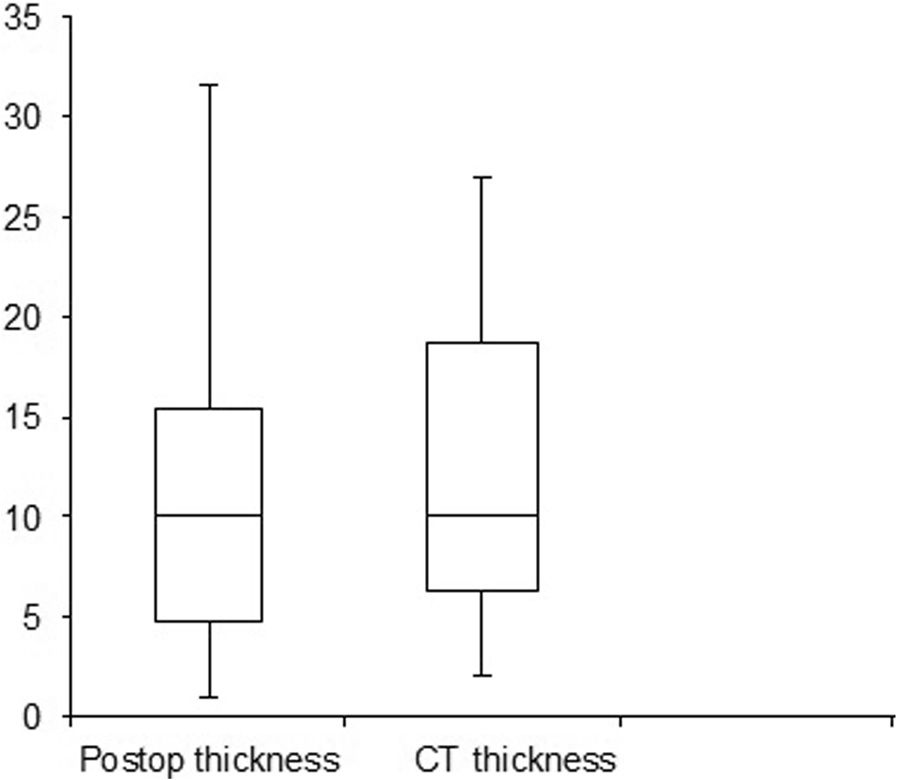
Shows the box and whisker box plot for CT scan based tumor thickness and postoperative tumor thickness

The mean postoperative final pathological tumor thickness was 11.60 mm while the mean CT tumor thickness was 12.88 mm. The correlation between CT thickness and postoperative thickness was highly significant (Spearman rho = 0.755, *P* < 0.001) (Fig. 1). Figure 2 shows the box and whisker box plot for CT scan based tumor thickness and postoperative tumor thickness.

## Discussion

In order to better stratify OSCC patients according to their occult cervical metastasis risk, growing evidence supports the use of tumor thickness [14]. It would be very useful to establish a reliable method to predict tumor thickness preoperatively, thus avoiding two-step surgery.

While there are currently no established methods of assessing tumor thickness pre-operatively, three main options are currently being investigated: preoperative biopsy, intraoral ultrasonography, and CT imaging.

To our knowledge, there is very limited data on the accuracy of preoperative tumor biopsy to predict histological tumor thickness. We previously conducted an unpublished retrospective study and compared tumor thickness estimated from preoperative tumor biopsy with the final postoperative pathologic measurement. We demonstrated a lack of significant correlation between the two measurements in thicker tumors and concluded that tumor thickness should not be reported in biopsy reports, as especially thicker tumors were at risk to harbour nodal metastasis.

There is emerging evidence that intraoral ultrasonography may accurately predict histological tumor thickness in OSCC. Shintani *et al*. were among the first to demonstrate this correlation in tongue carcinoma by comparing preoperative ultrasound estimation of tumor thickness with measurements obtained from postoperative histological sections [15]. Since then, many authors have published similar results with other sub sites (i.e. floor of mouth) while also showing direct correlation between ultrasound tumor thickness and neck metastasis [16, 17]. Limitations to intraoral ultrasonography include tenderness, trismus, or anatomic location hindering adequate ultrasound probe placement. Furthermore, discordances between pathological and ultrasound derived tumor thickness have been recorded in thicker tumors (i.e. >20 mm) and is explained by transducer limitation as well as tissue shrinkage secondary to histological processing [15, 16].

The use of imaging in assessing tumor thickness has not being discussed critically in the literature. Park *et al.* performed a retrospective study to evaluate the accuracy of MRI in measuring the invasion depth of different oral cavity cancers [18]. The authors calculated Pearson’s correlation coefficient for histologic and imaging invasion depths in oral tongue, tongue base, and tonsil cancers of 0.949, 0.941, and 0.578, respectively. Their study also demonstrated a direct correlation between radiologic invasion depth and lymph node metastasis in oral tongue and tongue base cancers with calculated invasion depth cut-off values of 9.5 mm and 14.5 mm, respectively. Interestingly, the mean invasion depth measured from histologic specimens was less than when measured by imaging. This is likely due to shrinking of tissue following resection and processing, which has been previously reported in the literature [19]. Lam *et al*. performed a similar study and validated the accuracy of imaging in estimating tumor thickness in oral tongue cancer [14].

On the other hand, Lwin *et al*. analyzed MRI imaging and histopathological reports of 102 consecutive OSCC cases and concluded that radiological staging of the neck or tumor thickness could not safely determine the need for neck dissection [20]. Indeed, the authors found no valid radiological tumor thickness threshold that could be used clinically to predict nodal involvement. Moreover, they reported a total of 11 tumors, with sizes varying from 2 mm to 24 mm, which were undetectable on radiology.

Our study results showed a significant correlation between postoperative final pathological tumor thickness and CT based tumor thickness. While MRI may be superior to CT in the evaluation of soft tissue lesions, our results may support the use of CT in measuring tumor thickness. This may be relevant as CT scanning is faster and usually more available than MRI.

In conclusion, this study supports a role for CT scan in determining tumor thickness in OTSCC.
